# 1,4-Bis[4-(meth­oxy­carbon­yl)benz­yl]-1*H*-1,2,4-triazol-4-ium bromide

**DOI:** 10.1107/S1600536812016728

**Published:** 2012-04-28

**Authors:** Wen-Jiao Guo, Hua-Rong Huang, Zhi-Yun Du, Yan-Xiong Fang, Kun Zhang

**Affiliations:** a Guangdong University of Technology, Faculty of Chemical Engineering and Light Industry, Guangzhou 510006, Guangdong, People’s Republic of China

## Abstract

In the title salt, C_20_H_20_N_3_O_4_^+^·Br^−^, the dihedral angle between the benzene rings is 8.69 (16)°, and those between the benzene rings and the triazole ring are 69.98 (18) and 72.17 (18)°. In the crystal, C—H⋯Br hydrogen bonds link the cations and anions into chains along the *c* axis.

## Related literature

For general background to triazole derivatives, see: Zanardi *et al.* (2011) ▶. For a related structure, see: Huang *et al.* (2010 ▶).
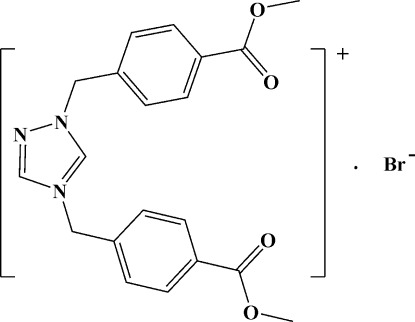


## Experimental

### 

#### Crystal data

C_20_H_20_N_3_O_4_^+^·Br^−^
*M* *_r_* = 446.30
Orthorhombic, 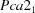

*a* = 33.6880 (15) Å
*b* = 4.7962 (3) Å
*c* = 12.3337 (6) Å
*V* = 1992.81 (18) Å^3^
*Z* = 4
Cu *K*α radiation
μ = 3.08 mm^−1^
*T* = 173 K
0.40 × 0.32 × 0.31 mm

#### Data collection

Oxford Diffraction Xcalibur Atlas Gemini Ultra diffractometer
Absorption correction: multi-scan (*ABSPACK* in *CrysAlis PRO*; Oxford Diffraction, 2006 ▶) *T*_min_ = 0.372, *T*_max_ = 0.449
6375 measured reflections
2709 independent reflections
2541 reflections with *I* > 2σ(*I*)
*R*_int_ = 0.027

#### Refinement

*R*[*F* ^2^ > 2σ(*F* ^2^)] = 0.027
*wR*(*F* ^2^) = 0.069
*S* = 1.05
2709 reflections
255 parameters
1 restraint
H-atom parameters constrained
Δρ_max_ = 0.37 e Å^−3^
Δρ_min_ = −0.24 e Å^−3^
Absolute structure: Flack (1983 ▶), 840 Friedel pairs
Flack parameter: 0.021 (18)

### 

Data collection: *CrysAlis PRO* (Oxford Diffraction, 2006 ▶); cell refinement: *CrysAlis PRO*; data reduction: *CrysAlis PRO*; program(s) used to solve structure: *SHELXS97* (Sheldrick, 2008 ▶); program(s) used to refine structure: *SHELXL97* (Sheldrick, 2008 ▶); molecular graphics: *SHELXTL* (Sheldrick, 2008 ▶); software used to prepare material for publication: *WinGX* (Farrugia, 1999) ▶ and *PLATON* (Spek, 2009 ▶).

## Supplementary Material

Crystal structure: contains datablock(s) global, I. DOI: 10.1107/S1600536812016728/ng5263sup1.cif

Structure factors: contains datablock(s) I. DOI: 10.1107/S1600536812016728/ng5263Isup2.hkl

Supplementary material file. DOI: 10.1107/S1600536812016728/ng5263Isup3.cml

Additional supplementary materials:  crystallographic information; 3D view; checkCIF report

## Figures and Tables

**Table 1:** Hydrogen-bond geometry (Å, °)

*D*—H⋯*A*	*D*—H	H⋯*A*	*D*⋯*A*	*D*—H⋯*A*
C1—H1⋯Br1	0.95	2.61	3.461 (3)	149
C3—H3*A*⋯Br1	0.99	2.91	3.795 (4)	149
C2—H2⋯Br1^i^	0.95	2.75	3.657 (3)	161

Symmetry code: (i) 
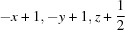
.

**Fig. 1.**
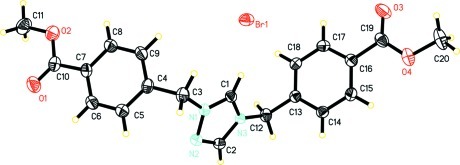
Perspective view showing 30% probability displacement ellipsoids and the atom-numbering scheme.

**Fig. 2.**
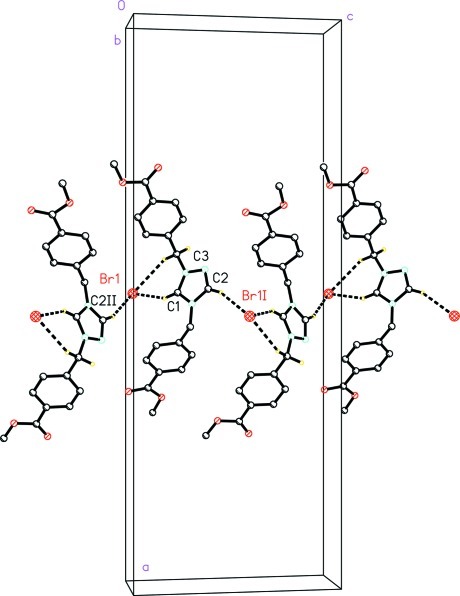
Crystal packing of the title compound. Dashed lines indicate hydrogen bonds. Symmetry: I = -x + 1, -y + 1, z + 1/2; II = -x + 1, -y + 2, z + 1/2. H atoms not involved in the hydrogen bond interactions have been omitted.

## supplementary crystallographic information

### Comment

1*H*-1,2,4-Triazole, like imidazole and benzimidazole, can be *N*-alkylated to form *N*-heterocyclic carbine (Zanardi *et al.*, 2011).

In an earlier report, we presented a *N*-heterocyclic benzimidazole derivative. The crystal structure consists of infinite chains connected *via* C—H···Br and C—H···O hydrogen bonds; it also shows π···π stacking interactions (Huang *et al.*, 2010).

In this work, we report the structure of a triazole-based *N*-heterocyclic carbene, 1,4-bis[4-(methoxycarbonyl)benzyl]-1*H*-1,2,4-triazolium bromide (Fig. 1).

In the crystal structure, the dihedral angles between the triazole ring system and the two (C4–C9) and (C13–C18) benzene rings are 69.98 (18) ° and 72.17 (18) °, respectively; the dihedral angle between the two benzene rings is 8.69 (16) °.

The bromide anions and cations form infinite hydrogen bonding chains along the *c*-axis *via* C—H···Br hydrogen bonds between the bromide anions and carbineen atoms of triazole ring and ethylene linkage (Fig. 2, Table 1).

### Experimental

4-(Methoxycarbonyl)benzyl bromide (2.28 g, 10.0 mmol) was slowly added to a solution of 1*H*-1,2,4-triazole (0.35 g, 5.0 mmol) in acetonitrile (25 ml) and the resulting mixture was stirred under reflux for 8 h.

The solvent was evaporated under reduced pressure. The solid residue was recrystallized in methanol, and colorless block crystals were obtained after few days, yield 1.42 g, 63.5%.

Elemental analysis, calcd (%) for C_20_H_20_N_3_BrO_4_: C 53.82, H 4.52, N 9.42; found(%): C 53.80, H 4.60, N 9.41.

The FAB mass spectrum showed tje ions at 447.

### Refinement

The C-bound H atoms were positioned geometrically and were included in the refinement in the riding-model approximation, with distances 0.98 (CH_3_), 0.99 (CH_2_) and 0.95 Å (aromatic and triazole); *U*_iso_(H) = *xU*_eq_(attached atom), where *x* = 1.5 for methyl C and 1.2 for all other C.

### Crystal data

**Table:**

C_20_H_20_N_3_O_4_^+^·Br^−^	*D*_x_ = 1.488 Mg m^−^^3^
*M**_r_* = 446.30	Cu *K*α radiation, λ = 1.5418 Å
Orthorhombic, *P**c**a*2_1_	Cell parameters from 3691 reflections
*a* = 33.6880 (15) Å	θ = 3.6–66.9°
*b* = 4.7962 (3) Å	µ = 3.08 mm^−^^1^
*c* = 12.3337 (6) Å	*T* = 173 K
*V* = 1992.81 (18) Å^3^	Block, colorless
*Z* = 4	0.40 × 0.32 × 0.31 mm
*F*(000) = 912	

### Data collection

**Table:**

Oxford Diffraction Xcalibur Atlas Gemini Ultra diffractometer	2709 independent reflections
Radiation source: Enhance Ultra (Cu) X-ray Source	2541 reflections with *I* > 2σ(*I*)
Mirror monochromator	*R*_int_ = 0.027
Detector resolution: 10.5058 pixels mm^-1^	θ_max_ = 66.9°, θ_min_ = 4.4°
φ and ω scans	*h* = −40→39
Absorption correction: multi-scan (ABSPACK in *CrysAlis PRO*; Oxford Diffraction, 2006)	*k* = −5→4
*T*_min_ = 0.372, *T*_max_ = 0.449	*l* = −11→14
6375 measured reflections	

### Refinement

**Table:**

Refinement on *F*^2^	Secondary atom site location: difference Fourier map
Least-squares matrix: full	Hydrogen site location: inferred from neighbouring sites
*R*[*F*^2^ > 2σ(*F*^2^)] = 0.027	H-atom parameters constrained
*wR*(*F*^2^) = 0.069	*w* = 1/[σ^2^(*F*_o_^2^) + (0.0373*P*)^2^ + 0.0837*P*] where *P* = (*F*_o_^2^ + 2*F*_c_^2^)/3
*S* = 1.05	(Δ/σ)_max_ < 0.001
2709 reflections	Δρ_max_ = 0.37 e Å^−^^3^
255 parameters	Δρ_min_ = −0.24 e Å^−^^3^
1 restraint	Absolute structure: Flack (1983), 840 Friedel pairs
Primary atom site location: structure-invariant direct methods	Flack parameter: 0.021 (18)

### Special details

**Table:**

Geometry. All e.s.d.'s (except the e.s.d. in the dihedral angle between two l.s. planes) are estimated using the full covariance matrix. The cell e.s.d.'s are taken into account individually in the estimation of e.s.d.'s in distances, angles and torsion angles; correlations between e.s.d.'s in cell parameters are only used when they are defined by crystal symmetry. An approximate (isotropic) treatment of cell e.s.d.'s is used for estimating e.s.d.'s involving l.s. planes.
Refinement. Refinement of *F*^2^ against ALL reflections. The weighted *R*-factor *wR* and goodness of fit *S* are based on *F*^2^, conventional *R*-factors *R* are based on *F*, with *F* set to zero for negative *F*^2^. The threshold expression of *F*^2^ > σ(*F*^2^) is used only for calculating *R*-factors(gt) *etc*. and is not relevant to the choice of reflections for refinement. *R*-factors based on *F*^2^ are statistically about twice as large as those based on *F*, and *R*- factors based on ALL data will be even larger.

### Fractional atomic coordinates and isotropic or equivalent isotropic displacement parameters (Å^2^)

**Table:**

	*x*	*y*	*z*	*U*_iso_*/*U*_eq_	
C1	0.47864 (8)	0.6564 (6)	0.2417 (3)	0.0375 (7)	
H1	0.4887	0.7596	0.1819	0.045*	
C2	0.47240 (9)	0.3718 (7)	0.3756 (3)	0.0452 (7)	
H2	0.4788	0.2350	0.4285	0.054*	
C3	0.40894 (9)	0.8422 (7)	0.2458 (3)	0.0480 (8)	
H3A	0.4184	0.9793	0.1917	0.058*	
H3B	0.3980	0.9465	0.3083	0.058*	
C4	0.37667 (8)	0.6644 (6)	0.1961 (3)	0.0441 (7)	
C5	0.34867 (10)	0.5364 (9)	0.2611 (3)	0.0554 (9)	
H5	0.3490	0.5665	0.3373	0.067*	
C6	0.32036 (11)	0.3655 (9)	0.2160 (3)	0.0599 (10)	
H6	0.3017	0.2741	0.2616	0.072*	
C7	0.31867 (9)	0.3249 (7)	0.1051 (3)	0.0450 (7)	
C8	0.34607 (8)	0.4583 (7)	0.0390 (4)	0.0538 (7)	
H8	0.3449	0.4351	−0.0374	0.065*	
C9	0.37509 (10)	0.6255 (8)	0.0851 (3)	0.0533 (9)	
H9	0.3941	0.7143	0.0399	0.064*	
C10	0.28761 (9)	0.1331 (7)	0.0608 (4)	0.0518 (10)	
C11	0.26013 (14)	−0.0757 (11)	−0.0946 (4)	0.0807 (13)	
H11A	0.2335	−0.0263	−0.0690	0.121*	
H11B	0.2662	−0.2681	−0.0735	0.121*	
H11C	0.2612	−0.0592	−0.1737	0.121*	
C12	0.53934 (10)	0.3639 (7)	0.2875 (3)	0.0456 (8)	
H12A	0.5389	0.2054	0.2361	0.055*	
H12B	0.5492	0.2936	0.3580	0.055*	
C13	0.56784 (9)	0.5850 (7)	0.2453 (3)	0.0389 (7)	
C14	0.59021 (9)	0.7440 (7)	0.3159 (3)	0.0421 (7)	
H14	0.5868	0.7241	0.3919	0.051*	
C15	0.61770 (9)	0.9330 (7)	0.2758 (3)	0.0454 (7)	
H15	0.6334	1.0398	0.3246	0.054*	
C16	0.62246 (8)	0.9668 (7)	0.1643 (3)	0.0428 (7)	
C17	0.59997 (10)	0.8069 (8)	0.0951 (3)	0.0521 (8)	
H17	0.6030	0.8288	0.0190	0.063*	
C18	0.57294 (9)	0.6139 (8)	0.1348 (3)	0.0475 (7)	
H18	0.5580	0.5021	0.0861	0.057*	
C19	0.65221 (11)	1.1610 (8)	0.1181 (4)	0.0541 (10)	
C20	0.70101 (11)	1.5049 (8)	0.1569 (5)	0.0781 (14)	
H20A	0.7055	1.6461	0.2130	0.117*	
H20B	0.6921	1.5960	0.0901	0.117*	
H20C	0.7258	1.4045	0.1429	0.117*	
N1	0.44256 (8)	0.6675 (5)	0.2823 (2)	0.0398 (6)	
N2	0.43770 (8)	0.4859 (7)	0.3659 (2)	0.0497 (8)	
N3	0.49824 (8)	0.4702 (6)	0.3018 (2)	0.0345 (5)	
O1	0.26382 (8)	0.0139 (6)	0.1165 (3)	0.0724 (8)	
O2	0.28892 (9)	0.1106 (6)	−0.0466 (3)	0.0679 (7)	
O3	0.65877 (9)	1.1836 (7)	0.0219 (3)	0.0767 (10)	
O4	0.67098 (7)	1.3101 (5)	0.1933 (3)	0.0648 (7)	
Br1	0.479859 (9)	1.16298 (6)	0.04187 (3)	0.04923 (11)	

### Atomic displacement parameters (Å^2^)

**Table:**

	*U*^11^	*U*^22^	*U*^33^	*U*^12^	*U*^13^	*U*^23^
C1	0.0373 (15)	0.0360 (17)	0.0391 (17)	−0.0023 (11)	0.0009 (12)	0.0032 (13)
C2	0.0461 (17)	0.051 (2)	0.0381 (17)	−0.0054 (13)	−0.0026 (13)	0.0080 (14)
C3	0.0408 (15)	0.0435 (19)	0.060 (2)	0.0051 (13)	−0.0021 (15)	−0.0033 (15)
C4	0.0349 (14)	0.0423 (18)	0.0550 (19)	0.0042 (11)	−0.0035 (14)	−0.0035 (15)
C5	0.0490 (18)	0.073 (3)	0.0444 (19)	−0.0087 (16)	0.0028 (15)	−0.0006 (17)
C6	0.0504 (18)	0.080 (3)	0.050 (2)	−0.0217 (17)	0.0059 (16)	0.0001 (18)
C7	0.0335 (14)	0.049 (2)	0.0527 (19)	0.0010 (12)	0.0005 (13)	−0.0019 (15)
C8	0.0473 (15)	0.070 (2)	0.0442 (15)	−0.0054 (13)	0.006 (2)	−0.003 (2)
C9	0.0431 (16)	0.066 (2)	0.0507 (18)	−0.0115 (15)	0.0083 (14)	0.0001 (16)
C10	0.0411 (14)	0.0516 (18)	0.063 (3)	0.0008 (13)	−0.0052 (17)	−0.0019 (19)
C11	0.083 (3)	0.087 (3)	0.072 (3)	−0.020 (2)	−0.022 (3)	−0.009 (3)
C12	0.0394 (16)	0.0411 (18)	0.056 (2)	0.0024 (13)	−0.0035 (16)	0.0029 (15)
C13	0.0333 (14)	0.0384 (17)	0.0450 (18)	0.0048 (12)	−0.0016 (13)	0.0034 (15)
C14	0.0405 (15)	0.0466 (17)	0.0392 (16)	0.0031 (12)	−0.0009 (13)	0.0001 (14)
C15	0.0393 (15)	0.0486 (19)	0.0483 (18)	−0.0006 (12)	−0.0026 (13)	−0.0061 (15)
C16	0.0367 (14)	0.0421 (18)	0.0494 (17)	0.0055 (12)	0.0017 (13)	0.0057 (14)
C17	0.0513 (18)	0.066 (3)	0.0386 (16)	−0.0032 (16)	−0.0007 (15)	0.0064 (16)
C18	0.0474 (17)	0.055 (2)	0.0405 (17)	−0.0066 (13)	−0.0061 (14)	−0.0029 (15)
C19	0.0439 (17)	0.051 (2)	0.068 (3)	0.0036 (14)	0.0061 (18)	0.0162 (19)
C20	0.0489 (19)	0.049 (2)	0.137 (4)	−0.0081 (16)	0.016 (2)	0.009 (2)
N1	0.0370 (12)	0.0420 (16)	0.0402 (14)	−0.0050 (10)	−0.0018 (12)	−0.0007 (11)
N2	0.0437 (14)	0.064 (2)	0.0408 (16)	−0.0075 (13)	0.0009 (12)	0.0105 (14)
N3	0.0365 (11)	0.0365 (14)	0.0304 (12)	−0.0044 (9)	0.0003 (9)	0.0020 (11)
O1	0.0623 (16)	0.082 (2)	0.0729 (19)	−0.0267 (15)	0.0071 (14)	−0.0048 (16)
O2	0.0654 (16)	0.0780 (19)	0.0603 (16)	−0.0199 (14)	−0.0075 (14)	−0.0071 (15)
O3	0.0720 (17)	0.087 (2)	0.071 (3)	−0.0134 (14)	0.0174 (17)	0.0213 (16)
O4	0.0499 (13)	0.0555 (16)	0.089 (2)	−0.0115 (10)	0.0067 (14)	0.0025 (15)
Br1	0.06480 (19)	0.04233 (17)	0.04057 (16)	−0.00337 (13)	−0.0031 (2)	0.0041 (2)

### Geometric parameters (Å, º)

**Table:**

C1—N1	1.316 (4)	C11—H11B	0.9800
C1—N3	1.335 (4)	C11—H11C	0.9800
C1—H1	0.9500	C12—N3	1.486 (5)
C2—N2	1.296 (4)	C12—C13	1.522 (5)
C2—N3	1.345 (4)	C12—H12A	0.9900
C2—H2	0.9500	C12—H12B	0.9900
C3—N1	1.479 (4)	C13—C18	1.381 (5)
C3—C4	1.512 (4)	C13—C14	1.381 (5)
C3—H3A	0.9900	C14—C15	1.387 (5)
C3—H3B	0.9900	C14—H14	0.9500
C4—C5	1.382 (5)	C15—C16	1.394 (5)
C4—C9	1.383 (5)	C15—H15	0.9500
C5—C6	1.376 (5)	C16—C17	1.376 (5)
C5—H5	0.9500	C16—C19	1.482 (5)
C6—C7	1.382 (5)	C17—C18	1.388 (5)
C6—H6	0.9500	C17—H17	0.9500
C7—C8	1.388 (5)	C18—H18	0.9500
C7—C10	1.497 (5)	C19—O3	1.212 (6)
C8—C9	1.386 (5)	C19—O4	1.331 (5)
C8—H8	0.9500	C20—O4	1.449 (4)
C9—H9	0.9500	C20—H20A	0.9800
C10—O1	1.200 (5)	C20—H20B	0.9800
C10—O2	1.331 (6)	C20—H20C	0.9800
C11—O2	1.445 (5)	N1—N2	1.359 (4)
C11—H11A	0.9800		
			
N1—C1—N3	105.8 (3)	C13—C12—H12A	109.0
N1—C1—H1	127.1	N3—C12—H12B	109.0
N3—C1—H1	127.1	C13—C12—H12B	109.0
N2—C2—N3	111.9 (3)	H12A—C12—H12B	107.8
N2—C2—H2	124.1	C18—C13—C14	119.9 (3)
N3—C2—H2	124.1	C18—C13—C12	119.1 (3)
N1—C3—C4	110.8 (3)	C14—C13—C12	120.9 (3)
N1—C3—H3A	109.5	C13—C14—C15	120.1 (3)
C4—C3—H3A	109.5	C13—C14—H14	120.0
N1—C3—H3B	109.5	C15—C14—H14	120.0
C4—C3—H3B	109.5	C14—C15—C16	120.3 (3)
H3A—C3—H3B	108.1	C14—C15—H15	119.9
C5—C4—C9	119.3 (3)	C16—C15—H15	119.9
C5—C4—C3	120.4 (3)	C17—C16—C15	119.0 (3)
C9—C4—C3	120.3 (3)	C17—C16—C19	118.9 (3)
C6—C5—C4	120.2 (3)	C15—C16—C19	122.0 (3)
C6—C5—H5	119.9	C16—C17—C18	120.9 (3)
C4—C5—H5	119.9	C16—C17—H17	119.5
C5—C6—C7	120.9 (3)	C18—C17—H17	119.5
C5—C6—H6	119.6	C13—C18—C17	119.8 (3)
C7—C6—H6	119.6	C13—C18—H18	120.1
C6—C7—C8	119.3 (3)	C17—C18—H18	120.1
C6—C7—C10	118.4 (3)	O3—C19—O4	123.2 (4)
C8—C7—C10	122.3 (3)	O3—C19—C16	123.8 (4)
C9—C8—C7	119.7 (4)	O4—C19—C16	113.0 (3)
C9—C8—H8	120.2	O4—C20—H20A	109.5
C7—C8—H8	120.2	O4—C20—H20B	109.5
C4—C9—C8	120.7 (3)	H20A—C20—H20B	109.5
C4—C9—H9	119.6	O4—C20—H20C	109.5
C8—C9—H9	119.6	H20A—C20—H20C	109.5
O1—C10—O2	123.6 (4)	H20B—C20—H20C	109.5
O1—C10—C7	123.4 (4)	C1—N1—N2	112.0 (3)
O2—C10—C7	113.0 (3)	C1—N1—C3	127.9 (3)
O2—C11—H11A	109.5	N2—N1—C3	120.1 (3)
O2—C11—H11B	109.5	C2—N2—N1	103.4 (3)
H11A—C11—H11B	109.5	C1—N3—C2	106.9 (3)
O2—C11—H11C	109.5	C1—N3—C12	128.6 (3)
H11A—C11—H11C	109.5	C2—N3—C12	124.2 (3)
H11B—C11—H11C	109.5	C10—O2—C11	115.8 (4)
N3—C12—C13	112.9 (3)	C19—O4—C20	117.5 (4)
N3—C12—H12A	109.0		
			
N1—C3—C4—C5	84.0 (4)	C14—C13—C18—C17	−1.3 (5)
N1—C3—C4—C9	−95.3 (4)	C12—C13—C18—C17	−177.5 (3)
C9—C4—C5—C6	2.0 (6)	C16—C17—C18—C13	1.3 (5)
C3—C4—C5—C6	−177.3 (3)	C17—C16—C19—O3	−1.9 (6)
C4—C5—C6—C7	−1.8 (6)	C15—C16—C19—O3	175.5 (4)
C5—C6—C7—C8	0.2 (6)	C17—C16—C19—O4	177.8 (3)
C5—C6—C7—C10	179.1 (3)	C15—C16—C19—O4	−4.9 (5)
C6—C7—C8—C9	1.2 (5)	N3—C1—N1—N2	−1.9 (4)
C10—C7—C8—C9	−177.6 (3)	N3—C1—N1—C3	179.8 (3)
C5—C4—C9—C8	−0.6 (6)	C4—C3—N1—C1	111.4 (4)
C3—C4—C9—C8	178.7 (3)	C4—C3—N1—N2	−66.8 (4)
C7—C8—C9—C4	−1.0 (5)	N3—C2—N2—N1	−0.8 (4)
C6—C7—C10—O1	0.6 (5)	C1—N1—N2—C2	1.7 (4)
C8—C7—C10—O1	179.4 (3)	C3—N1—N2—C2	−179.8 (3)
C6—C7—C10—O2	−180.0 (4)	N1—C1—N3—C2	1.3 (4)
C8—C7—C10—O2	−1.1 (5)	N1—C1—N3—C12	175.9 (3)
N3—C12—C13—C18	−90.6 (4)	N2—C2—N3—C1	−0.3 (4)
N3—C12—C13—C14	93.2 (4)	N2—C2—N3—C12	−175.2 (3)
C18—C13—C14—C15	0.1 (5)	C13—C12—N3—C1	35.4 (5)
C12—C13—C14—C15	176.2 (3)	C13—C12—N3—C2	−150.9 (3)
C13—C14—C15—C16	1.1 (5)	O1—C10—O2—C11	−1.6 (6)
C14—C15—C16—C17	−1.1 (5)	C7—C10—O2—C11	178.9 (3)
C14—C15—C16—C19	−178.4 (3)	O3—C19—O4—C20	−0.9 (5)
C15—C16—C17—C18	−0.1 (5)	C16—C19—O4—C20	179.4 (3)
C19—C16—C17—C18	177.3 (3)		

### Hydrogen-bond geometry (Å, º)

**Table:**

*D*—H···*A*	*D*—H	H···*A*	*D*···*A*	*D*—H···*A*
C1—H1···Br1	0.95	2.61	3.461 (3)	149
C3—H3*A*···Br1	0.99	2.91	3.795 (4)	149
C2—H2···Br1^i^	0.95	2.75	3.657 (3)	161

Symmetry code: (i) −*x*+1, −*y*+1, *z*+1/2.

## References

[bb7] Farrugia, L. J. (1999). *J. Appl. Cryst.* **32**, 837–838.

[bb1] Flack, H. D. (1983). *Acta Cryst.* A**39**, 876–881.

[bb2] Huang, H.-R., Wen-Jiao, G., Du, Z.-Y., Fang, Y.-X. & Zhang, K. (2010). *Acta Cryst.* E**66**, o3064. 10.1107/S1600536810041929 PMC3011811 21589374

[bb3] Oxford Diffraction (2006). *CrysAlis PRO.* Oxford Diffraction Ltd., Abingdon, England.

[bb4] Sheldrick, G. M. (2008). *Acta Cryst.* A**64**, 112–122. 10.1107/S0108767307043930 18156677

[bb5] Spek, A. L. (2009). *Acta Cryst.* D**65**, 148–155. 10.1107/S090744490804362X PMC2631630 19171970

[bb6] Zanardi, A., Mata, J. A. & Peris, E. (2011). *Eur. J. Inorg. Chem.* pp. 416–421.

